# Depression-Biased Reverse Plasticity Rule Is Required for Stable Learning at Top-Down Connections

**DOI:** 10.1371/journal.pcbi.1002393

**Published:** 2012-03-01

**Authors:** Kendra S. Burbank, Gabriel Kreiman

**Affiliations:** 1 Department of Neurology and Ophthalmology, Children's Hospital Boston, Harvard Medical School, Boston, Massachusetts, United States of America; 2 Center for Brain Science, Harvard University, Cambridge, Massachusetts, United States of America; 3 Swartz Center for Theoretical Neuroscience, Harvard University, Cambridge, Massachusetts, United States of America; University of Oxford, United Kingdom

## Abstract

Top-down synapses are ubiquitous throughout neocortex and play a central role in cognition, yet little is known about their development and specificity. During sensory experience, lower neocortical areas are activated before higher ones, causing top-down synapses to experience a preponderance of post-synaptic activity preceding pre-synaptic activity. This timing pattern is the opposite of that experienced by bottom-up synapses, which suggests that different versions of spike-timing dependent synaptic plasticity (STDP) rules may be required at top-down synapses. We consider a two-layer neural network model and investigate which STDP rules can lead to a distribution of top-down synaptic weights that is stable, diverse and avoids strong loops. We introduce a temporally reversed rule (rSTDP) where top-down synapses are potentiated if post-synaptic activity precedes pre-synaptic activity. Combining analytical work and integrate-and-fire simulations, we show that only depression-biased rSTDP (and not classical STDP) produces stable and diverse top-down weights. The conclusions did not change upon addition of homeostatic mechanisms, multiplicative STDP rules or weak external input to the top neurons. Our prediction for rSTDP at top-down synapses, which are distally located, is supported by recent neurophysiological evidence showing the existence of temporally reversed STDP in synapses that are distal to the post-synaptic cell body.

## Author Summary

The complex circuitry in the cerebral cortex is characterized by bottom-up connections, which carry feedforward information from the sensory periphery to higher areas, and top-down connections, where the information flow is reversed. Changes over time in the strength of synaptic connections between neurons underlie development, learning and memory. A fundamental mechanism to change synaptic strength is spike timing dependent plasticity, whereby synapses are strengthened whenever pre-synaptic spikes shortly precede post-synaptic spikes and are weakened otherwise; the relative timing of spikes therefore dictates the direction of plasticity. Spike timing dependent plasticity has been observed in multiple species and different brain areas. Here, we argue that top-down connections obey a learning rule with a reversed temporal dependence, which we call reverse spike timing dependent plasticity. We use mathematical analysis and computational simulations to show that this reverse time learning rule, and not previous learning rules, leads to a biologically plausible connectivity pattern with stable synaptic strengths. This reverse time learning rule is supported by recent neuroanatomical and neurophysiological experiments and can explain empirical observations about the development and function of top-down synapses in the brain.

## Introduction

Connectivity patterns between different areas in neocortex are often discussed in terms of bottom-up and top-down connections [1], . With few exceptions, communication between any two connected neocortical areas occurs in both directions [1], [4]. Feedforward or “bottom-up” connections are those which run from lower neocortical areas (such as visual area V1) to higher areas (such as V2); they typically but not always originate in layers 2/3 and synapse onto neurons in layer 4 [1], [4], . By contrast, feedback or “top-down” connections, which run from higher neocortical areas to lower ones, typically originate in layer 6 and frequently synapse onto distal dendrites in layer 1. While bottom-up synapses have been widely studied and modeled, the development, functions and properties of the more-abundant top-down connections are less well understood [2], [3], [7].

Here we investigate the learning rules that govern the development of top-down connections in neocortex. We study variations on a classical paradigm describing changes in synaptic strength between two neurons: spike-timing dependent plasticity (STDP) [8], [9], [10]. According to STDP, when a pre-synaptic spike occurs within tens of milliseconds before a post-synaptic spike, the synaptic strength is enhanced. Conversely, when a pre-synaptic spike occurs shortly after a post-synaptic spike, the synaptic strength is decreased. STDP has been observed in a wide variety of systems and conditions [11] and has been examined in many computational studies as well (e.g. [12], [13], [14]; for reviews, see [15], [16], [17]).

**Figure 1:**
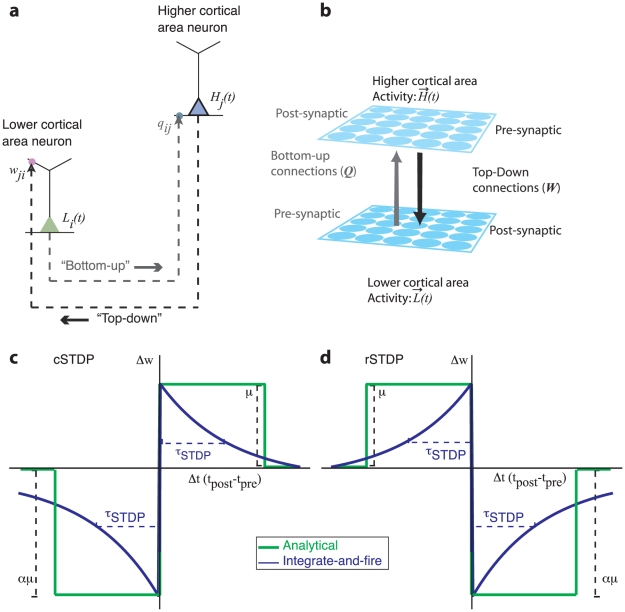
Schematic description of the model and learning rules. **a**. Schematic description of the model used in the analytical and computational work. The model consists of two layers: a “lower” cortical area (units with activity *L_i_*(t)) and a “higher” cortical area (units with activity *H_j_(t)*). **b**. The strength of the all-to-all bottom-up connections from the lower area to the higher area is represented by the matrix **Q** (gray arrows). These synapses occur in proximal dendrites and their weights are fixed unless otherwise noted. The strength of the all-to-all top-down connections from the higher area to the lower area is represented by the matrix **W** (black arrows). These synapses occur in distal dendrites. The **W** weights evolve according to the plasticity rules described in **c–d**. There are no connections within each layer. **c**. Schematic description of “classical” spike-time dependent plasticity (cSTDP). For a given synapse, the y-axis indicates the change in the weight (*Δw*) and the x-axis represents the temporal difference between the post-synaptic action potential and the pre-synaptic action potential (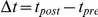). The green curve shows the learning rule used in the analytical section while the blue curve shows the learning rule used in the integrate-and-fire simulations. In cSTDP, a pre-synaptic action potential followed by a post-synaptic action potential (*Δt>0*) leads to potentiation (*Δw>0*). The learning rate at each synapse is controlled by the parameter *μ* and the ratio of depression to potentiation is controlled by *α*. In the computational simulations, the parameter *τ_STDP_* controls the rate of weight change with *Δt*. **d**. Schematic description of “reverse” STDP (rSTDP).

In order to calculate the effects of different types of learning rules in neocortical circuits, the relative timing of firing events during signal propagation needs to be taken into account. During activity evoked by transient stimuli, neurons in a lower area such as V1 will generally be activated before neurons in a higher area (such as V4) [18], [19], [20], [21]. Under this scenario, bottom-up synapses will experience a predominance of pre-synaptic spikes followed by postsynaptic ones (“pre-post” spike pairs). For top-down synapses, on the other hand, the identities of the pre- and post-synaptic neurons are reversed, meaning that stimulus-evoked activity will be experienced as a predominance of “post-pre” spike pairs. Here, motivated by this identity reversal, we hypothesize that the learning rule at top-down synapses might exhibit unusual temporal dependences. Specifically, we propose that learning at top-down synapses follows a temporally reversed version of spike-time-dependent plasticity, which we call rSTDP (**Figure 1**).

We compare the long-term effects of training a population of top-down synapses using either classical STDP (cSTDP) or rSTDP. We argue that the plasticity rules must lead to a distribution of top-down synaptic weights that fulfills the following three key properties. (1) *Top-down weights should be stable*. When the statistics describing the environment are stationary, the top-down connections should settle into an unchanging pattern, allowing the information carried through top-down connections to be consistently interpreted. (2) *Top-down weights should be diverse*. We expect to observe a continuous distribution of strengths in top-down connections with a significant spread (as opposed to binary weights or all weights taking the same value) [14]. Functionally, a diverse set of top-down connections can perform a richer set of computations. (For discussions of the computational properties of synapses with graded strengths, see [22], [23].) (3) *Top-down weights should be weak*. Specifically, top-down connections should not create any strong loops [15], [24], [25], as these can amplify neuronal activity to pathological levels. We emphasize that this condition does not preclude the existence of strong *individual* top-down connections; these are permitted so long as the combined effect of all bottom-up and top-down connections does not lead to runaway excitation.

Using analytical methods and numerical simulations, we compare networks whose top-down connections exhibit plasticity via rSTDP with those whose top-down connections exhibit classical STDP. We further examine the effects of biasing learning towards depression or towards potentiation. We argue that depression-biased rSTDP, but not cSTDP, can lead to a stable, diverse and weak distribution of top-down weights. Finally, we show that the model's predictions are consistent with recent experimental findings about the relationship between plasticity and neuroanatomy.

## Results

We study the characteristics of synaptic plasticity learning rules at top-down synapses and evaluate whether the resulting distribution of synaptic strengths fulfill the three properties outlined above: stability, diversity and weakness. We start by considering a simple model that we can solve analytically, and then evaluate the results with integrate-and-fire simulations. The network models described in the analytical and integrate-and-fire sections share the same basic structure (**Figure 1a–b**). The model consists of two levels of neurons, with every neuron in the lower level connected reciprocally to every neuron in the higher level. A number of simplifications should be noted: (i) there are no lateral connections within a level; (ii) there is no separation of excitatory and inhibitory neurons although weights can take positive or negative values; (iii) external inputs arrive only at the lower level (except in section “**Top-down modulatory signals”**).

The steps in each computational experiment were to a) generate a network with initial bottom-up and top-down synaptic strengths; b) specify an external stimulus to initiate activity in lower-level neurons; c) calculate the resultant neuronal activity over time in the network; d) change synaptic weights according to this activity and to our specified learning rule; and e) repeat steps b–d until we can determine the characteristics of the final weight distribution. In most cases, we modify only the top-down weights in step (d), keeping the bottom-up weights constant (however, we explore concurrent modification of bottom-up weights and top-down weights in the section “**Additional stability mechanisms**”.) The outcomes of this basic paradigm are calculated analytically in the first section and determined through simulations in the integrate-and-fire section.

### Analytical model of plasticity at top-down synapses

In this section, we consider model neurons whose activities at each time-point are linear sums of the synaptic inputs at the previous time-point. During a stimulus presentation, the activity in lower-level neurons at the first time-point 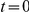 is given by 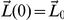, where 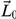 is a vector describing the external inputs to the lower-level neurons. Activity in higher-level neurons in the next time-point is 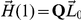, where 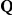 is a matrix describing bottom-up synaptic weights. Activity then propagates back to the lower-level neurons, with 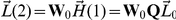, where 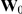 is the matrix of top-down weights. We assume that plasticity is slow, so that we can approximate top-down weights as unchanging during a single stimulus presentation (see **Text S1**). Activity continues to move up and down through the network during the stimulus presentation.

At the end of each stimulus presentation, we determine the change in synaptic strength for each pair of neurons by considering the joint activities of those two neurons, as calculated in every pair of adjacent time-points during a stimulus presentation. Because we focus on the top-down synapses, the higher-level units are pre-synaptic and the lower-level units are post-synaptic. The learning rule is a simplified version of spike-timing dependent plasticity (STDP) (**Figure 1c–d** and **Text S1**). The learning rule is written here for clarity with two terms: the first term represents joint activity from events when the post-synaptic lower-level units are activated *before* the pre-synaptic higher-level units (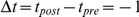), while the second term describes joint activity from events when the post-synaptic lower-level units are activated *after* the pre-synaptic higher-level units (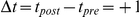). We write two equations, one describing cSTDP (**Figure 1c** **,** **Eq 1**) and the other one describing rSTDP (**Figure 1d** **,** **Eq 1′**):
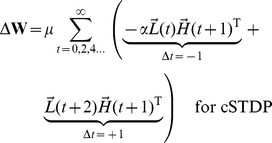
(1)

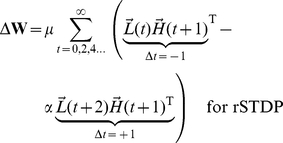
(1′)


The learning rate of synaptic plasticity is set by a parameter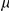. The parameter describing the balance between depression and potentiation is 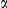; when 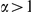, depression dominates over potentiation. **Equation 1** reflects cSTDP, in which the weights increase from joint activity where 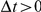 and decrease when 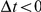 (**Figure 1c**). The alternative learning rule considered here (rSTDP) is given by **Equation 1′**; in this case the weights decrease from joint activity when 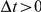 and increase when 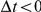 (**Figure 1d**). As discussed below, this sign reversal between cSTDP and rSTDP is at the heart of the discussion about the stability of the learning rule for top-down synapses.

Using the expressions for neuronal activity and synaptic plasticity, we can determine and characterize *fixed points* of the system. These are sets of top-down weights which produce activity that, on average, leads to no further change in the weights. Fixed points represent potential places where the weights might settle after multiple stimulus presentations. To find an expression for fixed points, we plug the expressions for neuronal activity into **Equation 1** or **1′** and look for points where Δ**W** becomes zero (**Text S1**).

We show that any fixed point 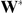 must obey a simple relation: for cSTDP, the relation is 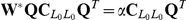; for rSTDP, it is 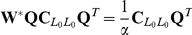. Here, 
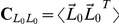
 is the cross-correlation matrix formed by averaging the joint initial activity of pairs of lower-level neurons across many external stimuli. These equations imply that for cSTDP 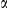 is an eigenvalue of 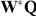, and that 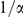 is an eigenvalue of 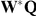 for rSTDP.

From these relations, we see that for both cSTDP and rSTDP, top-down weights at a fixed point will typically be *diverse*: they will make up a continuous distribution, and will not be binary or single-valued. Counter-examples exist only for very particular choices of 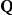 and 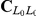, such as when the distribution of bottom-up weights 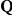 is itself single-valued. We conclude that fixed points in this model will generally meet the criterion of diversity, regardless of the parameters of the learning rule.

We also note that potential fixed points depend both on the bottom-up weights (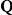) and on the statistical structure of the external inputs (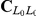). The presence of the correlation term 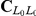, specifically, means that we can describe the learning rule as *correlative*. In the special case where 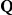 is invertible, the relations simplify to 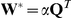
for cSTDP and 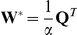
for rSTDP, meaning that top-down connections simply reproduce a scaled version of earlier lower-level activity (see further discussion below.)

### Requirements for prevention of strong loops

We ask whether top-down weights at fixed points meet the criterion of weakness (defined as the absence of strong excitatory loops.) A strong loop exists whenever there are patterns of neuronal activity which are amplified as they pass up and down through the network. Because the network is linear, activity at any time-point can be calculated by multiplying the previous activity by the matrix **W_0_Q** (for example 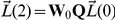.) The activity will increase, implying the existence of strong loops, whenever the matrix 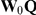 has eigenvalues greater than one. As discussed above, for fixed points, 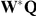 has eigenvalues of 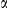 (for cSTDP) and 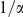
(for rSTDP) (see **Text S1** for further details). This means that strong loops must exist at every fixed point for depression-biased cSTDP and for potentiation-biased rSTDP. Thus, the only plasticity rules which can produce weak and potentially stable top-down weights are potentiation-biased cSTDP and depression-biased rSTDP.

### Depression-biased reverse STDP is required for development of unchanging top-down weights

Finally, we consider the requirement for stability. We evaluate whether fixed points are stable or not by performing a linear stability analysis, which examines the effect of plasticity when weights are close to but not equal to a fixed point. If the fixed point is stable, plasticity must draw the weights ever closer; if it is unstable, plasticity will push weights away from the fixed point.

To perform the stability analysis, we calculate how the difference between the current top-down weights and the fixed point changes over time [26] (**Text S1**). We show that under cSTDP, at least one component of the difference between the current top-down weights and the fixed point will actually *grow* over time as a result of plasticity, and hence the fixed point must be *unstable*. Therefore, in the model architecture presented here, networks where top-down connections are trained with cSTDP cannot have any stable fixed points. By contrast, networks in which top-down connections are learned with rSTDP may have stable fixed points. We conclude that fixed points in this model can meet the criterion of stability only for rSTDP. Putting these results together, we see that only for depression-biased rSTDP can plasticity lead to sets of top-down weights that simultaneously meet the criteria of stability, diversity and weakness.

An intuitive understanding of the requirement for rSTDP can be gained by considering only the first three time-points in a stimulus presentation. The top-down weights only affect activity starting at time *t = 2*. For cSTDP, the pre-post synaptic joint activity from times 1 and 2 leads to potentiation and increased activity at *t = 2*, which in turns causes further potentiation. In this positive feedback loop the weights can increase indefinitely. By contrast, with rSTDP, joint activity from times 1 and 2 leads to depression. Any increase in the strength of the top-down weights will cause more activity at *t = 2* and thus lead to additional depression, bringing the weights back into balance. Therefore these circuits will tend to self-stabilize. The analytical work discussed above and in the **Text S1** together with the simulations in the next section formalize and extend this argument beyond the initial time points.

We emphasize that the requirement for rSTDP only applies to learning at top-down synapses. A similar analysis can be performed for bottom-up synapses by holding 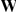 constant while modifying 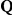. In the **Text S1**, we show for simple cases that stable training of bottom-up synapses requires cSTDP. Therefore, the results presented here are consistent with the existence of a conventional plasticity rule (cSTDP) at bottom-up connections while implying the necessity of a temporally reversed plasticity rule (rSTDP) for top-down connections. Concurrent changes in **W** and **Q** are considered in section **“Additional stability mechanisms”**.

We also considered the case where the lower and upper cortical areas were not reciprocally connected. In the **Text S1**, we show that rSTDP is still required at top-down connections in this case. Because the mathematics in this case are somewhat simpler, we were able to move beyond linear neurons and show that the requirement for rSTDP holds when the neurons have an arbitrary non-linear but monotonic activation function. We also show that in this case the bias towards depression is not necessary, since strong excitatory loops cannot develop in the absence of reciprocal connections.

### Example of development of top-down weights

**Figure 2:**
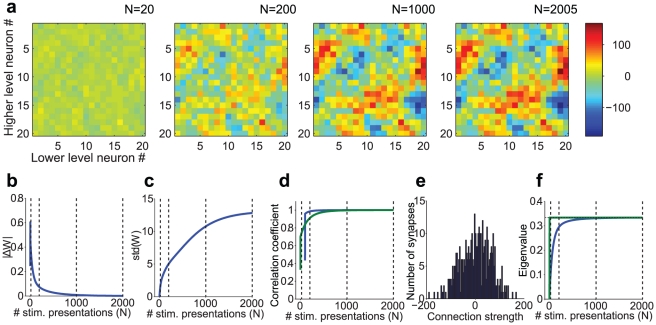
Example numerical implementation of the analytical results for depression-biased rSTDP learning. **a.** Development of top-down synaptic weights (**W**) over multiple stimulus presentations. *N* indicates the stimulus presentation number and here we show 4 snapshots of **W** *(N)*. This model had 20 lower units and 20 higher units. The strength of each synaptic weight is represented by the color in the **W** matrix (see scale on the right). The algorithm converged after 2005 iterations and the final **W** is shown on the right (see Methods for convergence criteria). **b–d.** Measures of weight stability and diversity. **b.** Norm of the change in the top-down weight matrix (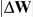) as a function of stimulus presentation number *N* (see text). As the algorithm converges, the change in the weights becomes smaller. The dotted lines mark the iterations corresponding to the snapshots shown in part **a**. **c.** Standard deviation of the distribution of top-down weights as a function of iteration presentation number (loosely represented in the y-axis as std(**W**)). The final value in this plot (*N* = 2005) corresponds to the standard deviation of the distribution shown in part **e**. **d.** Pearson correlation coefficient between the vectorized **W** *(N)* and **W**(*N-100*) (blue line, calculated only for *N> = 100*) and between **W**(*N*) and the predicted value of **W** at the fixed point (W* = **Q^−1^**; green line, see text for details). As the algorithm converges, **W** *(N)*→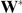. **e.** Measure of weight diversity: Distribution of the final synaptic weights after the algorithm converged. Bin size = 4. **f**. Measure of absence of strong loops: Mean (blue) and maximum (green) eigenvalue of the matrix **WQ**, as a function of stimulus presentation number. This matrix describes the activity changes produced in a full up-down loop through the network. Eigenvalues greater than one would correspond to the existence of strong loops. The maximum eigenvalue never surpasses 0.33, which is equal to 1/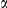. The mean eigenvalue also eventually stabilizes at this value.

As a sanity check and to illustrate the dynamical changes in the weights as a consequence of the learning rule, we created a numerical implementation of our analytical network by using **Equation A22** (see **Text S1**). **Figure 2** shows the results of a simulation with rSTDP and depression dominating (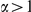). The evolution of the top-down weight matrix **W** over multiple stimulus presentations is shown in **Figure 2a**. The weights change rapidly at the beginning and converge to a stable solution. The magnitude of the changes in **W** approaches zero as the algorithm converges (**Figure 2b**) and the standard deviation of the weights approaches a constant value (**Figure 2c**). We predicted that the top-down weight matrix **W** would approach the inverse of 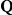 when 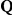 is invertible, as it is in **Figure 2**. **Figure 2d** shows that the correlation coefficient between **W** and 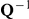 indeed approaches 1 over time. The final distribution of weights is continuous and diverse (as opposed to being binary or single-valued) (**Figure 2e**). Finally, all the eigenvalues of **W** are below 1 (**Figure 2f**) as required to avoid runaway excitation. In sum, we have illustrated that the circuit simulated in **Figure 2** fulfills the three requisite criteria: the final distribution of weights is stable, diverse and does not lead to runaway excitation.

### In an integrate-and-fire simulation, depression-biased rSTDP leads to stable, diverse and weak top-down weights

We supplement the analytical results above by relaxing many of the assumptions and simulating a network under biologically more realistic conditions. We performed numerical simulations of a network of noisy and leaky integrate-and-fire neurons (**Eqs. 2** **–** **5**, **Methods**). The simulations differed in four key ways from the analytical work above. First and most importantly, the integrate-and-fire model neurons enabled us to better simulate the non-linear responses that neurons typically display in response to their inputs as well as explicitly include spikes and plasticity rules based on spike timing (**Eqs. 2** **–** **4**). Second, the external input to lower-level neurons occurred over an extended period of time. Third, instead of considering only adjacent pairs of spikes we used a plasticity rule which varied smoothly in strength depending on the time difference between pre- and post-synaptic spikes (**Eq. 5**). Fourth, we introduced noise into our simulations in the form of noisy synaptic inputs. In principle, these differences could lead to qualitatively different effects on the requirement for depression-biased rSTDP.

**Figure 3:**
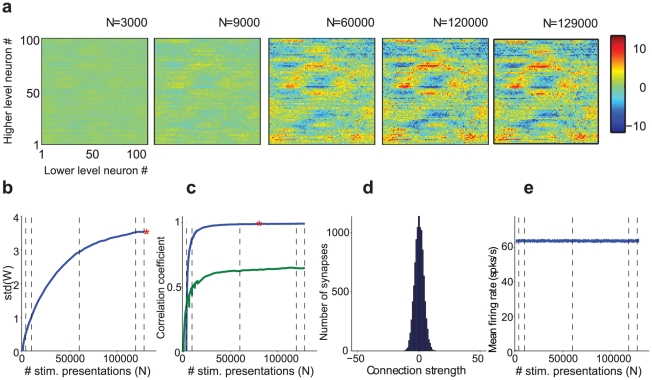
Example of the dynamics and evolution of top-down weights in the integrate-and-fire model. **a.** Snapshots showing the evolution of **W** *(N)* in the integrate-and-fire network simulations over time defined by the number of stimulus presentations (*N*). The format is the same as in **Figure 2a**. This model had 100 lower units and 100 higher units. The parameters used in this simulation are shown in the last column of **Table 1**, with rSTDP and 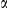 = 1.2. **b–c.** Measures of weight stability. **b**. Standard deviation of the distribution of top-down weights as a function of the stimulus presentation number. The convergence criterion for the standard deviation was that the slope of this plot (calculated as 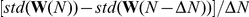 with *ΔN* = 6000) be less than 10^−5^. The convergence criterion was met at the point indicated by the red asterisk. The dotted vertical lines correspond to the times of the five snapshots shown in part **a**. **c.** Blue line: Pearson correlation coefficient between the vectorized ***W*** *(N)* and ***W*** *(N-ΔN)*, for Δ*N* = 3000 iterations. For comparison with **Figure 2**, we also show the correlation coefficient between **W**(*N*) and the inverse of **Q** (green line). We note that in the integrate and fire simulations we do not expect **W**(*N*) to converge to the 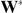
described in the text and **Figure 2**. A simulation run was classified as ‘convergent’ when the correlation coefficient was greater than 0.99 and when the std criterion in part **b** was met. In this example, the simulation achieved the correlation criterion at *T* = 75000 (red asterisk). **d**. Measure of weight diversity: Distribution of the synaptic weights for the final snapshot. Bin size = 0.1. **e**. Measure of absence of strong loops: Average firing rate for lower-level neurons as a function of stimulus presentation number. The average firing rate almost immediately stabilizes to a constant value, and does not increase to pathological levels as occurs in the presence of strong excitatory loops.

**Figure 4:**
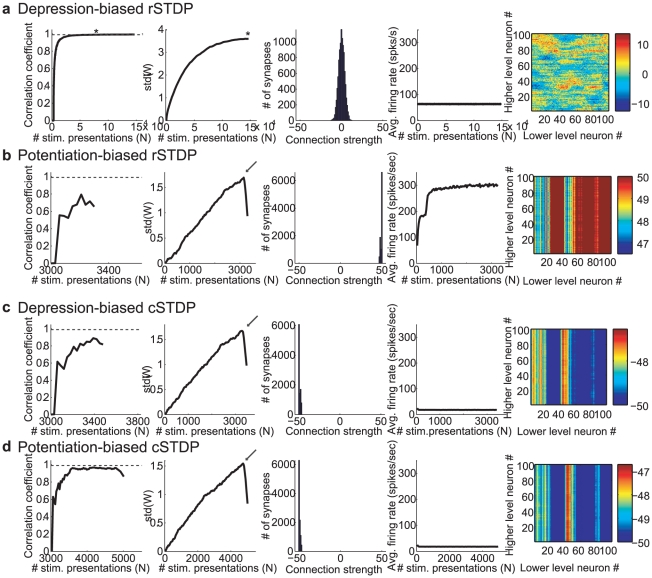
Representative results of integrate-and-fire simulations for different learning rules. We consider here four possible learning rules: classical STDP (cSTDP, **c**,**d**), reverse STDP (rSTDP, **a**,**b**), depression-biased (**a**,**c**) and potentiation-based learning (**b**,**d**). For each learning rule, we show the results for a representative simulation (see summary results in **Figure 5** **.**) The format and conventions for the subplots are the same as in **Figure 3**. The subplots show the Pearson correlation coefficient between the vector containing all the entries of **W** *(N)* and that for **W** *(N-ΔN)*, for ΔN = 3,000 iterations (first subplot), the standard deviation of the distribution of weights (second subplot), the distribution of weights (third subplot), the average firing rate of the lower level units (fourth subplot) and the final **W**. The simulation in part **a** converged; the convergence criteria were met at the value of *N* indicated by an asterisk. The simulations in **b–d** were classified as having “extreme weights” meaning that >50% of the weights were either at 0 or at the weight boundaries (±50). The arrows in the second subplot in **b–d** denote inflection points where the weights reached the boundaries and the standard deviation started to decrease. The parameters for each of these simulations are listed in the last column of **Table 1**, with specifics as follows. **a** rSTDP, 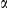 = 1.2; **b**: rSTDP, 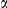 = 0.9; **c**: cSTDP, 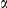 = 1.2; **d**: cSTDP, 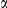 = 0.9. For the simulations in **b–d**, the weights varied most strongly across lower-level neurons, leading to the appearance of vertical bands in the final subplots (note the differences in the color scale and standard deviation values in **4b–d** compared to **4a**). Some lower-level neurons experienced greater joint activity than others due to the choice of **Q** (and hence greater plasticity); the instability of learning in these simulations then magnified these initial imbalances.

Apart from these differences, the model used in the simulations was similar to the one used in the analytical formulation (**Figure 1**). Bottom-up connections were fixed (and generated as in **Figure 2a**). The external inputs to each lower-level neuron were drawn from Gaussian distributions as described in **Methods**. Top-down weights were initially set to zero. In **Figure 3**, we show the evolution of top-down weights in one example simulation in which we used depression-biased rSTDP. In **Figure 4**, we show typical results of these simulations for each of the four main possible learning rules.

**Figure 5:**
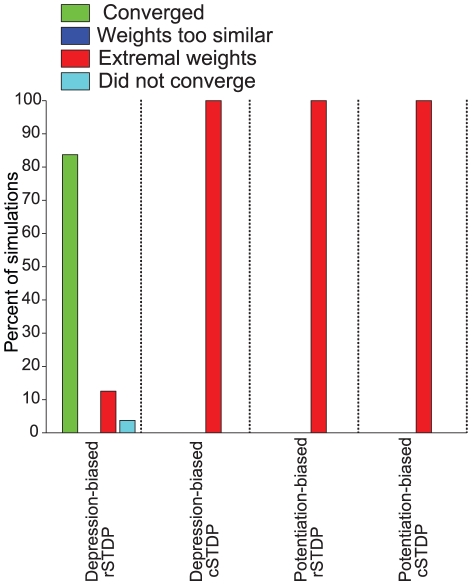
Summary of the results of the integrate-and-fire network simulations. We consider the four possible learning rules illustrated in **Figure 4**. Here we show the proportion of all the computational simulations in a parameter search (**Methods**, **Table 1**) using integrate-and-fire units that converged (green), that reached extreme weights (red) or that did not converge (light blue). For comparison with **Figure 6**, we included a category for simulations in which weights failed to achieve sufficient diversity (dark blue), although none of the current simulations fell into that category. The quantitative criteria for classifying the stimulations into these four categories as well as the network and parameters spanned are described in the text. The total number of simulations for each learning rule were 2298, 2304, 1148, and 1152. The only convergent simulations were seen for depression-biased rSTDP.

Each simulation was classified with one of four possible outcomes (see **Methods** for details). The first of these outcomes was “converged”; in order to qualify, a simulation's final top-down weights needed to satisfy our three key criteria of stability, diversity, and weakness. We assessed stability by calculating the cross-correlation of the current weights with those from previous time-points (**Figure 3c** **,** **4a** first subplot) as well as comparing the standard deviation of current and past weight distributions (**Figure 3b** **,** **4a** second subplot). We assessed diversity by asking whether the standard deviation of the top-down weights, when the simulation was stopped, surpassed a threshold value of 0.3 (**Figure 3d**, **4a** third subplot). We ensured that weights had not become too strong, assessing the absence of strong loops, by requiring that a convergent simulation have less than 50% of its weights at the maximum or minimum allowed weight. Simulations not labeled as “Converged” were categorized as “Weights too similar”, “Extreme weights”, or – in the rare cases when weights had not stabilized after 625,000 stimulus presentations – “Did not converge” (**Figure 4b–d**),

We next asked how the results of the simulations illustrated in **Figure 3** and **4a** depended on the parameters used in the simulations. In particular, we asked whether convergence required depression-biased rSTDP as it did for the linear network. We ran 6912 simulations, spanning a wide range of different sets of parameters as outlined in **Table 1**, including two bottom-up weight matrices 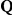 and two external stimulus correlation matrices 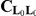. We ran each simulation three times with different initial conditions. We summarize the results of this parameter landscape characterization in **Figure 5**. Among the simulations with depression-biased rSTDP, convergence did not require fine-tuning of parameters – more than 90% of the simulations were categorized as convergent. Critically, none of the simulations with any of the other learning rules (potentiation biased rSTDP, potentiation or depression biased cSTDP) led to convergent simulations. Thus, in spite of the differences from the analytic work, the integrate-and-fire network simulations also lead us to a requirement for depression-biased rSTDP to achieve a stable, diverse and weak distribution of top-down weights.

**Table 1:** Parameters used in the integrate-and-fire simulations.

Parameter	Description	Values explored for Figure 5 and 7	Values explored for Figures 3, 4, 6, 8
	Learning type	rSTDP, cSTDP	rSTDP, cSTDP for Figs. 3, 4, 6. rSTDP for Fig. 8.
*A*	Depression/Potentiation balance	0.9, 1.2, 3	0.9,1.2 for Figs. 3, 4, 6. 0.9 for Fig. 8.
*D*	Synaptic transmission delay	1, 15 ms	15 ms
*τ_STDP_*	STDP time constant	5, 10, 20 ms	20 ms
*S*	Noise level	1000, 2000 spikes/sec	2000 spikes/sec
*τ_syn_*	Synaptic time constant	5, 15 ms	15 ms
*σ_input_*	Input variance	100%,200%	100%
*σ_noise_*	Noise variance	100%, 200%	50%
*W _min_, W _max_*	Minimum/maximum weight	50 for Figs. 3,4,6; 20 in Fig. 5	50
*F*	Target firing rate, for homeostatic scaling	n/a	n/a for Figs. 3–4, 8; 20,80,120 spikes/sec for Fig. 6
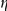	Relative strength of learning, for homeostatic scaling	n/a	n/a for Figs. 3–4, 8; 0.1,1,10,100 for Fig. 6
*ζ*	Relative strength of learning, for concurrent plasticity	n/a	n/a for Figs. 3–4, 8; −10,−1,−0.1, 0.1,1,10 for Fig. 6

Only parameters that were varied are shown in here; for other parameters that were fixed across simulations, see Methods.

### Additional stability mechanisms

We have to this point considered only networks with pure STDP-type plasticity at top-down connections, and we have shown that cSTDP is unstable in these networks. We now modify the basic plasticity rule from **Equation 1** in one of several ways – by considering concurrent changes in bottom-up weights, by adding homeostatic synaptic scaling [27] or by using a multiplicative STDP rule [28], [29], [30], [31]. These last two mechanisms have been shown to stabilize inherently unstable Hebbian learning in feedforward networks [31] and recurrent networks [28], [32]. However, this stabilization can cause a loss of synaptic competition [14], [28]. We asked how our conclusions would be affected by adding these mechanisms. For each of these mechanisms, we modified the linear firing-rate model (**Methods**) and evaluated the systems numerically and using our integrate-and-fire model (**Methods**).

**Figure 6:**
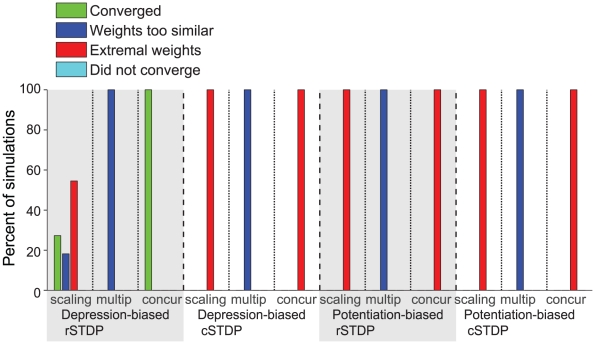
Summary of the results of the integrate-and-fire network simulations with additional stability mechanisms. We show the results of simulations with homeostatic scaling, multiplicative plasticity, or concurrent bottom-up and top-down plasticity (**Methods**, **Table 1**). The format is the same as in **Figure 5**. The only convergent simulations were seen for depression-biased rSTDP, in the homeostatic scaling and concurrent plasticity cases. For all other learning conditions, homeostatic scaling simulations and concurrent plasticity reached extreme weights. Multiplicative plasticity always led to a lack of diversity.

In homeostatic synaptic scaling, all incoming synapses to a given neuron are modified simultaneously so as to help a neuron maintain a target firing rate. To model this homeostatic mechanism, we first applied the weight changes predicted by STDP, then multiplied all top-down connection weights and external inputs to a given neuron by a factor that depended on the difference between the current firing rate and the target firing rate (**Methods**). First, we tested homeostatic scaling with the depression-biased rSTDP learning rule in our linear model. We confirmed that, as in the case without synaptic scaling, these networks generally converged to stable, diverse and weak distributions of weights. Next, we considered *potentiation*-biased rSTDP learning rules, which were unstable in the non-scaling case. We found that with homeostatic synaptic scaling, although learning did sometimes acquire stable and weak connection weights, the distributions were never diverse: the standard deviations of the weights was always at least 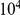 times smaller than those in the depression-biased cases. Finally, we looked at cSTDP learning rules, with either potentiation or depression biases. We found no combinations of parameters in which homeostatic synaptic scaling with cSTDP led to stable and diverse top-down weights. We confirmed each of these results using integrate-and-fire simulations: homeostatic synaptic scaling only allowed for convergent behavior with depression-biased STDP, and led to extreme weights or loss of diversity in every other case (**Table 2**, **Figure 6**). In several depression-biased rSTDP simulations, the pull towards homeostasis was enough to shift the steady-state weight values high enough that a fraction of the feedback weights moved into the “extreme” range, causing more simulations to be labeled as “extreme weights” than in the case without homeostasis (**Figure 5**); however, learning was not truly unstable in these cases.

**Table 2:** Summary of results for modifications to the plasticity rules described in **Equation 1**.

**Homeostatic Scaling**	*Potentiation bias*	*Depression bias*
*rSTDP*	Stable but not diverse/EW	**Stable and diverse**/**C**, WTS, EW
*cSTDP*	Unstable/EW	Unstable/EW
**Multiplicative Scaling**	*Potentiation bias*	*Depression bias*
*rSTDP*	Unstable/WTS,	Stable but not diverse/WTS
*cSTDP*	Unstable/WTS	Unstable/WTS
**Concurrent changes in Q**	*Potentiation bias*	*Depression bias*
*rSTDP*	Unstable/EW	**Stable**/**C**
*cSTDP*	Unstable/EW	Unstable/EW

We considered three modifications: Homeostatic scaling, Multiplicative scaling and concurrent changes in the bottom-up and top-down weights (see text for a description of these modified rules). The first line in each entry of the table describes the results of the numeric implementation of the analytical work (based on **Equation A22**). The second line of each entry describes the results of the Integrate and Fire simulations, by listing all the different outcomes seen for a particular modification. **C** = “Converged”, WTS = “Weights too similar”, EW = “Extreme Weights” (see main text and Methods for a quantitative definition of each of these categories).

Another modification of STDP used in several studies is a multiplicative learning rule [28], [29], [30] in which the change in a synaptic weight depends both on the current value of that weight and on amounts of pre- and post-synaptic activity. Here, we consider the particular implementation used in [30], in which the strength of potentiation is linearly proportional to the distance between the current weight and a maximum weight, while the strength of depression is proportional to the distance of the current weight from zero (**Eq. 6** **–** **7** **,** **Methods**). To test the effects of multiplicative scaling in our linear model, we modified **Equation A22**, for several values of the maximum weight (**Methods**), and we tested learning rules with rSTDP or cSTDP and 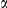 greater than or less than 1. In every case, all of the synaptic weights eventually clustered tightly at single values either close to zero or close to the maximum weight: we lost all diversity in the synaptic weights, analogous to a loss of synaptic competition. We therefore conclude that multiplicative learning is insufficient to allow for the development of stable and diverse synaptic weights under either cSTDP or potentiation-biased learning. The results were similar in the integrate-and-fire simulations: every simulation was classified as “weights too similar” (**Table 2** **,** **Figure 6**).

We argue that the loss of diversity under multiplicative scaling is due to the quadratic nature of the multiplicative learning rule (**Eq 6** **–** **7** **,** **Methods**), in which **W** appears explicitly and multiplies 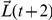, which implicitly depends on **W**. Quadratic learning rules will tend to be bi-stable, with fixed-point weights either very strong (near the maximum allowed value) or very weak (near zero). This binary weight pattern has indeed been observed in fully recurrent networks trained with a multiplicative cSTDP rule [32]; we interpret the results of our simulations as feedback weights clustering at the stronger of the two potential fixed points. Binary learning of this sort can create a new functional connectivity within a network; for instance, it can lead to the reduction of loops [33]. However, it is not a satisfactory solution here because we require diversity in the top-down weights.

The results presented thus far have assumed that the bottom-up weights remain unchanged and that there is plasticity only in the top-down weights. We evaluated whether the results would change when bottom-up connections were allowed to change concurrently with the top-down connections. We started with a set of randomly determined set of bottom-up weights 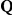 (**Methods**), but we now allowed **Q** to change over time with a learning rule analogous to that in **Equation 1** (**Eq. 8**). We considered all combinations of cSTDP and rSTDP as well as depression versus potentiation bias for plasticity (16 possible combinations). For both the numerical implementation of the linear work and for the integrate-and-fire simulations, we found convergent learning only when top-down connections were trained with depression-biased rSTDP (**Table 2** **,** **Figure 6**). Stability did not depend critically on the parameters of bottom-up learning; we found stable examples for bottom-up plasticity both with cSTDP and with rSTDP and with both depression and potentiation biases. We observed that the fraction of convergent simulations was increased relative to the case with no bottom-up plasticity (**Figure 5**). This improvement and further variations on simultaneous bottom-up and top-down learning deserve further study in future work.

### Top-down modulatory signals

Until this point, we have assumed that external input to the system arrives in the form of initial activity in the lower layer. This is a good way of modeling the bottom-up flow of information that might be expected to dominate during sensory-driven activity (e.g. flashes of visual stimuli). However, it is clear that top-down signals modulate and transform inputs as they arrive (e.g. [2], [34], [35], [36]). We asked whether and how such additional external input to the top layer impacts the stabilizing effects of rSTDP.

**Figure 7:**
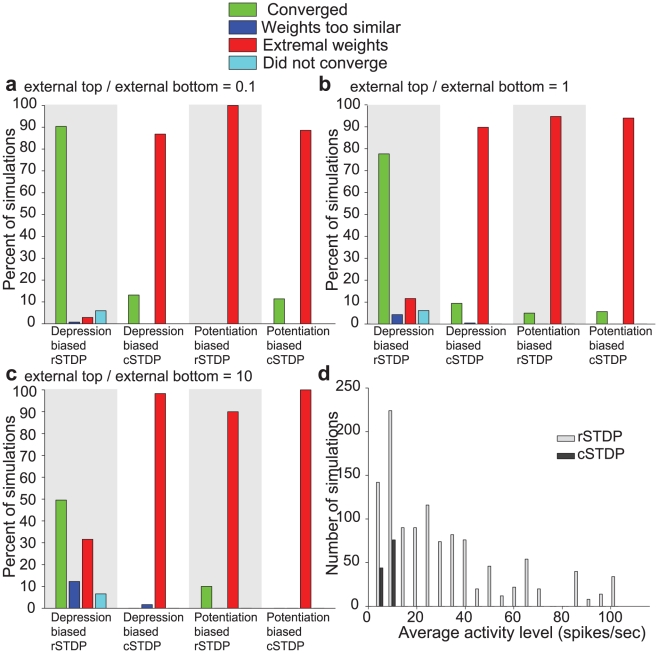
Summary of the results of the integrate-and-fire simulations with external input to bottom-layer and higher-layer neurons. **a–c.** In the simulations described here, external input was conveyed both to lower-level neurons and to higher-level neurons. The ratio of the external input strength to higher-level neurons to lower-level neurons was 0.1 in **a**, 1 in **b** and 10 in part **c**. The format and other parameters are the same as in **Figure 5**. **d.** For those simulations that converged (green in parts **a–c**), the histogram shows the distribution of average activity levels. The gray bars denote simulations using rSTDP and the dark bars denote simulations using cSTDP. Results from all three strength ratios (**a–c**) are combined in this plot. Those few simulations which are convergent under cSTDP have very low average firing rates.

We ran integrate-and-fire and numerical simulations using the same parameters from **Figure 5**, with the addition of simultaneous external input to the top layer (**Methods** **,** **Figure 7**). We considered different possible scenarios where the external input to the top units could be stronger (10 times), equal or weaker (1/10) than the external input to the bottom units. We found that depression-biased rSTDP was still able to generate sets of top-down weights which were stable, diverse, and weak (**Figure 7a–c**). When the external input to the top neurons was very strong (arguably a biologically less realistic condition [2], [7]), there were fewer simulations that converged, corresponding to a more restricted set of parameter values (**Figure 7c**). We also observed several simulations which met our convergence criteria even for cSTDP or potentiation bias. However, neurons in these simulations exhibited significantly less activity than in the depression-biased rSTDP case (**Figure 7d**). These cases constitute examples of a trivial fixed-point with low-activity levels where small amounts of potentiation and depression from higher and lower-layer external inputs cancel each other out.

### Computational significance of rSTDP learning

We have focused thus far on the requirements to make learning at top-down connections stable, diverse, and weak. These properties are necessary regardless of the computational role of top-down connections in any particular brain area. We now take initial steps towards considering the computations performed by the top-down connections after training in the particular architecture studied here. For linear neurons, we look at the fixed points of the training algorithm. As shown above, when the feedforward weight matrix **Q** is invertible, the fixed point 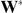 is 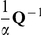. This means that after training, top-down connections create a scaled reconstruction of the initial lower-level neuronal activity. We show in the Text S1 that this principle applies even when **Q** is not invertible (so that perfect reconstruction is not always possible); in this case, the rSTDP learning rule minimizes the reconstruction error defined as the square of the difference between the input and its reconstruction.

**Figure 8:**
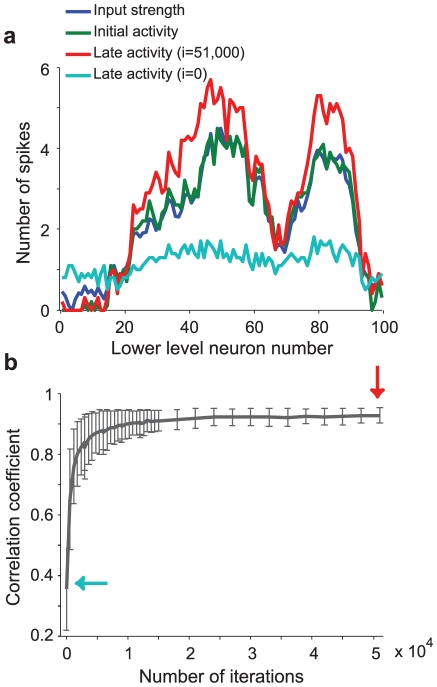
Integrate-and-fire network trained with rSTDP learns to reconstruct its input. **a.** Example of the network's ability to reconstruct its inputs after training using depression-biased rSTDP. By construction, the strength of external input during a single stimulus presentation to each neuron in the lower layer (input strength, blue line) is similar to the average spike rate of each lower-level neuron during the initial period from 0–50 ms (initial activity, green line). The cyan and red lines show the average spike rate of each lower-level neuron during the later period (late activity, 80–160 ms), when activity is due to top-down stimulation, using the top-down weights given early in training (after 10 iterations, cyan line) or after 51,000 iterations (red line). **b.** Average correlation coefficient between early time and late-time neuronal activity rates as a function of the number of training iterations. The average is computed over n = 100 distinct external input stimuli, and the error bars represent the standard deviation of the correlation coefficients for the 100 stimuli. The arrows indicate the iteration numbers illustrated in part **a**.

For networks of integrate-and-fire neurons, the picture is slightly more complicated. Frequently, the final **W** is well correlated with **Q** ^−1^ (e.g. **Figure 3c**). However, because of the non-linear nature of these neurons, the input strength is not always simply related to the amount of subsequent firing; in certain parameter regions, the input strength has more effect on the timing of neuronal firing than on the overall rate. We therefore focus on a regime where overall input is weak, so that only neurons with stronger inputs were able to fire. We did this by subtracting a constant value from the feedforward weight matrix **Q** used in previous sections (**Methods**). Under these conditions, we observed that after training with depression-biased rSTDP, the effect of the resulting top-down connections is to recreate an approximation to a scaled version of the original input (**Figure 8**). In **Figure 8a**, we show an example of how the network, after training, is capable of reconstructing a given activity pattern. The input to each lower-layer neuron (blue line) causes an early bout of activity in the lower-layer neurons (green line). Later in the stimulus presentation, the lower-level activity is due to feedback via the top-down connections. When the top-down weights have not yet been trained, this activity bears little resemblance to the initial activity (cyan line). However, after training is completed, the activity pattern constitutes a good reconstruction of the original input (red line). This effect is quantified in **Figure 8b**, which shows an increase in the correlation between early time and late-time neuronal activity as a function of the number of training iterations.

## Discussion

We studied plasticity at top-down synapses in a model of two reciprocally connected neocortical areas, such as visual areas V1 and V2. The strength of top-down synapses evolved according to an activity-dependent STDP-type learning rule. We asked which plasticity rules lead to a distribution of top-down weights which met three criteria: stability, diversity, and weakness (lack of strong loops). We studied this biological model analytically and using computer simulations, and we concluded that top-down synapses could achieve these three criteria only when their strength was governed by a depression-biased temporally-reversed STDP rule, rSTDP. By contrast, both classical STDP (cSTDP) and potentiation-biased rSTDP led to pathological outcomes such as the uncontrolled growth of synaptic weights or run-away neuronal excitation.

Under a temporally reversed STDP learning rule, post-synaptic spikes shortly followed by pre-synaptic spikes lead to potentiation and pre-synaptic spikes shortly followed by post-synaptic spikes lead to depression (**Figure 1d**). Our theoretical prediction for this type of temporal dependency is consistent with recent empirical evidence documented in several experimental systems (for a review, see [37]). In slices of rat visual cortex, pre-synaptic activity followed by post-synaptic activity caused synaptic depression while post-synaptic activity followed by pre-synaptic activity induced potentiation in distal L2/3 to L5 and L5 to L5 synapses [38]. A similar effect was observed in rat barrel cortex, where pairing single EPSPs with subsequent postsynaptic bursts induced depression at L2/3 to L5 distal synapses, while potentiation was induced when the timing was reversed [39]. Importantly, rSTDP has been observed only in *distal synapses* whereas cSTDP has been observed in synapses near the soma.

The neuroanatomical location of top-down synapses suggests that they are ideal candidates to display this temporally reversed form of synaptic plasticity: anatomical work shows that top-down connections occur predominantly at distal synapses [1], [4]. For example, tracing studies show that the synapses from visual area V2 to visual area V1 end up forming synaptic connections in the distal dendrites of layer 1 in V1 [6].

We considered depression and potentiation biased versions of STDP through the parameter *α* (**Figure 1c–d**). We found that a potentiation bias can lead to runaway excitation. Several experiments in different systems have found biases towards depression [38], [39], [40], [41], [42] (see however [9], [10]). A depression bias was also discussed and implemented in computational studies (e.g. [13], [14]).

Throughout most of our study, bottom-up connections were fixed to focus on the development of top-down connections because experimental studies suggest that bottom-up synapses may mature earlier than their top-down counterparts [43], [44]. However, in **Figure 6** we consider concurrent plasticity at bottom-up and top-down connections and show that this does not change our requirements for rSTDP at top-down synapses. We emphasize that we do *not* expect plasticity at bottom-up synapses to require rSTDP. Indeed, in the **Text S1** we show a case in which bottom-up synapses were only stable when trained with cSTDP, which is consistent with experimental evidence showing cSTDP at these synapses.

Critical to the analysis presented here was our choice of three criteria for successful plasticity: weights need to be stable, diverse and weak. What support can be found experimentally for the idea that top-down weights in biological neural networks exhibit these three properties? With regards to stability, there is evidence that many dendritic and axonal structures in adult cortex are stable over long periods of time yet change dramatically upon large changes to the sensory environment (for a review, see [45]).

The degree of diversity in top-down connections remains poorly understood at the experimental level. Some evidence implies that top-down synapses can connect neurons with different tuning preferences [5], [46] (but see also [47]), which might seem to be consistent with a generic, modulatory role for top-down signals, not requiring any particular diversity of synaptic weights. However, variations in synaptic weights occurring within the context of a broad non-selective connectivity pattern [48] could provide a mechanism for specificity of these signals. Several computational models that aim to describe the functions of top-down connections implicitly or explicitly assume a high degree of specificity (see e.g. [35], [36]).

We define weak distributions of top-down connections as those which keep the network from exhibiting any strong loops. It has long been recognized that strong loops must be avoided in cortical circuits [15], [24], [25], as these can amplify neuronal activity to pathological levels.

Our results depended crucially on several features in our biological model. First among these was our focus on *top-down* synapses (in contrast to bottom-up synapses which may require cSTDP). The second important feature was the timing of neuronal activity. We modeled each stimulus presentation as a flow of activity affecting first lower area and then higher area neurons; this initial bottom-up direction of flow was crucial for determining the effects of our timing-based learning rules. Different timing patterns could affect our results, an effect which we briefly explored in **Figure 7**. Third, the requirement for a depression bias in the learning rule arose because the cortical areas in our model were reciprocally connected, allowing for neuronal activity to reverberate up and down through the network. It is only in this context that activity can build up to pathological levels when strong loops exist.

Several other features in our model did not prove to be crucial to our results. For instance, reciprocal connectivity between the two cortical areas was *not* necessary in order for top-down connections to require rSTDP. In the **Text S1**, we showed that rSTDP is still required in a case where higher-level neurons are activated independently of lower level neurons, even for neurons with non-linear activation functions. (The external higher-level input in this case could be the result of a separate path, as in thalamic input feeding into both V1 and V2, or it could be a simplified description of a complicated multi-synaptic feedforward path between the two areas.) Similarly, none of our results depend on reciprocal connections between any two individual neurons.

Furthermore, rSTDP still led to adequate solutions in cases where modulatory external input to the top layer was added (**Figure 7a–b**). When the external input to the top layer was 10 times stronger than the external input to the bottom layer a smaller fraction of tested parameter values led to adequate solutions (**Figure 7c**). Biological data seems to suggest that external input to the top units would have a modulatory role consistent with the values in **7a** or even **7b** rather than **7c** [2]–[4], [7]. Yet, the results in 7c suggest that the stability of depression-biased rSTDP may show a stronger dependence on the particular parameter values when strong external input to the top layer is present compared to the situation when weaker external input to the top layer is present. We expect the effects of external stimuli to the top layer and bottom layer to differ given the asymmetry in our model imposed by changing **W** while maintaining **Q** fixed in **Equation 1**.

Our results also did not appear to depend on the exact form of the STDP learning rule. We used two different forms in our analytical and integrate-and-fire work (see **Figure 1**), including a variety of parameters in the integrate-and-fire case (**Table 1**), and additionally examined modifications including homeostatic scaling and multiplicative plasticity (**Figure 6**). In every case, the requirement for rSTDP was unchanged. Yet, while we have considered several possible modifications, we cannot rule out the existence of additional biological mechanisms that could help stabilize the network. For example, recent elegant work has shown that temporal shifts in the STDP rule also lead to stable and diverse solutions [14]. It is interesting to point out that in the vicinity of 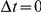 and on one side of the STDP learning rule, the net effect of the modifications introduced in [14] are similar to the ones we propose here.

Our integrate-and-fire simulations allowed us to relax many of the biologically unrealistic simplifications made in our analytical work. The simulations allowed us to make a better approximation of the complex nonlinear firing dynamics of real biological neurons, including synaptic transmission delays and noise. The results of these simulations are concordant with the analytical predictions and were robust to changes in many of the parameters in the simulations (**Table 1**, **Figure 5**) as well as different choices for the fixed bottom-up connection weights. Although simulations cannot exhaustively sample the entire parameter space, the parameter landscape described here in combination with the analytical work suggest the generality of the conclusions. Thus, we argue that our results may be relevant in biological circuits.

Using analytical work and integrate-and-fire simulations, we explored the computational significance of the rSTDP learning rule by showing that the network could learn to reconstruct its inputs (**Figure 8**). When the bottom-up weight matrix is orthogonal, the learning rule used here can lead to symmetric bottom-up and top-down weight matrices, which are known to show interesting computational properties (e.g. [35], [49]). A symmetric matrix also implies specificity in top-down modulatory signals as assumed in several computational models [7], [34], [35], [36]. Input reconstruction is closely related to “predictive coding” models [35], in which top-down information flow carries a prediction about subsequent lower-level activity. Predictive coding models also include the calculation of an error signal, which is the *difference* between the predicted and the actual activity; implementation of this error signal would presumably require the inclusion of populations of inhibitory neurons. It is intriguing to note that our rSTDP model does calculate exactly the required top-down signal for predictive coding. Another possible function for reconstructive signals is in the area of error correction. Suppose that the feedforward connections **Q** have been selected (or trained) with a method such as Principal Component Analysis (PCA) or Independent Component Analysis (ICA), so that the activity of the higher-layer neurons is a projection of lower-level activity which retains functionally important information while discarding irrelevant or noisy components. Then the reconstruction, given by the feedback connections, may be a de-noised version of the original input (e.g. [50]). This is also the principle used in de-noising autoencoders [51].

Ultimately, we hope that the hypothesis of reversed temporal dependence for plasticity at top-down synapses will be evaluated at the experimental level. The recent neurophysiological findings of temporal variations in STDP give experimental support for the existence of rSTDP at synapses which have distal dendritic locations, as top-down synapses do. Combining these findings with our computational results, we predict that a learning rule similar to rSTDP will be found to govern plasticity in top-down synapses in neocortex.

## Methods

### Numerical simulations of the analytical work

We considered a two-layer linear model that we can study analytically (**Text S1**). We illustrated the dynamical weight changes in this linear model by numerically simulating a network with 20 lower-area neurons and 20 higher-area neurons (**Figure 1a–b**). Each lower-area neuron was connected reciprocally to every higher-area neuron (but some of the weights could be zero). Although weights could be positive or negative, in the interest of simplicity and to reduce free parameters, we did not separate neurons into excitatory and inhibitory ones. The bottom-up weight matrix 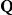 was chosen manually at the onset and was fixed (i.e. **Q** did not evolve according to plasticity rules) unless noted otherwise (**Figure 6**). Because we expected our final top-down weights **W** to be dependent on the inverse of **Q**, we wanted 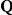 to be a well-conditioned random matrix. We generated it using the following algorithm: (i). Generate a uniformly distributed random matrix 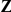, the same size as the desired **Q.** In some cases, for visualization purposes, smooth 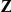 using a circular Gaussian filter of width 3 pixels. (ii). Calculate the polar decomposition of 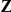 by finding unitary matrix 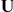
and positive semi-definite matrix 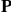 such that 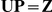. (iii). Calculate 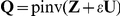. (iv). Normalize **Q** by dividing each column by its mean, then dividing the matrix by its maximum value and multiplying by 5. For the simulation in **Figure 2**, we did include the smoothing step and we set *ε* = 0.1. The top-down weights **W** were initialized to random, normally distributed values. **W** evolved according to the plasticity rule in **Equation A22**.

We stopped the simulations when either one of three conditions was reached: 1. If the matrix 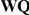 had any eigenvalues greater than one, we stopped the simulation and classified the outcome as ‘Extreme Weights’. 2. If the standard deviation of the weights was less than 10% of the initial value, we stopped the simulation and classified the outcome as ‘Too similar’. 3. When the standard deviation of the weights had stopped changing and the average weight changes became small and constant in magnitude, we classified the simulation as ‘converged’. At each time point, we considered the previous 50 stimulus presentations, and computed the average values and slopes for the standard deviation of the top-down weights and the changes in weights. We then required that (i) the slopes for the standard deviation and the weight changes be less than 0.1% of their respective average values, and (ii) either the average change in weights was less than 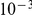 or the slope of the change and weights was smaller than the initial change in weights.

### Integrate-and-fire simulations

The architecture was the same as that for the numerical simulations of the analytical work, described above, except that each layer of the network contained 100 neurons. The nature of the numerical simulations created some additional differences to the analytical work. Our use of fixed time-steps (1 ms) ensured that there was a maximum firing rate that neurons could ever attain; we also imposed upper and lower limits on the values that top-down weights could attain (**Table 1**). Pathological scenarios which would cause activity or weights to become infinite in the analytical model would, in simulations, cause the firing rates or synaptic weights to reach their maximum allowed values. These constraints were not expected to affect network behavior in the cases where weights achieve an unchanging and diverse distribution, which were those that concerned us here.

The bottom-up weights **Q** were chosen as for the numerical simulation of the analytical work and were fixed unless otherwise noted. For the simulations in **Figures 3** and **4**, we included the smoothing step and set *ε* = 1. For the simulations in **Figures 5** and **6**, we did not include the smoothing step and we set *ε* = 0.1. We generated the initial top-down weights **W** as a uniformly distributed random matrix whose values ranged from −0.05 to 0.05.

Each lower level neuron's membrane potential *V_i_* evolved according to 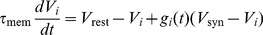
(2)with 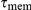 a membrane time constant of 10 ms, *V_rest_* = −74 mV, and 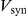 = 0 mV. Parameters whose values are not specified here were varied during the course of experiments; see **Table 1**. The neuron fired an action potential when its membrane potential reached 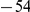
mV; when this occurred, the membrane potential was reset to −60 mV. 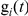 was a conductance determined by the incoming spikes that have occurred since neuron *i* fired its last action potential according to:
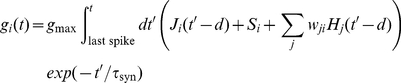
(3)where, 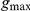 = 0.04. *J_i_(t)* is the rate of incoming external spikes due to the stimulus, and *d* is a synaptic transmission delay. *S_i_* is the number of spikes corresponding to excitatory noise, and its value is chosen randomly at each time-point from a Gaussian distribution of mean *S* spikes/sec and standard deviation equal to 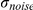 times the mean. *w_ji_* is the synaptic top-down weight connecting neuron *j* to neuron *i*, and *H_j_(t)* is 1 if higher-level neuron *j* fired an action potential at time *t* and 0 otherwise. Finally, 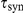 is a synaptic time constant. Higher-level neurons evolve according to a similar rule, except that they do not receive external stimulus input, so we have
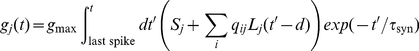
(4)where *L_i_(t)* and *q_ij_* represent the lower-level action potentials and bottom-up weights, respectively. We simulated the above dynamics using time-steps of 1 ms.

At the beginning of our simulations, we created a random cross-correlation matrix 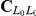. Then, for each stimulus presentation, we randomly generated a vector 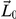 describing the strength of external input to each lower-level neuron, chosen such that their average cross-correlation when calculated across many stimulus presentations 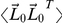 was equal to 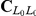. Within every stimulus presentation, the input strength *J_i_(t)* was chosen at each time-point from a Gaussian distribution with mean 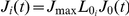 and standard deviation equal to 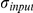 times the mean. 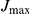 was 20,000 spikes/sec. *J_0_(t)* describes the time evolution of the input. It was the combination of an initial transient in the form of a Gaussian of height 1 centered at 30 ms with a width of 20 ms, followed by a sustained tonic input at 1/5 the maximum height that lasted for an additional 80 ms.

The synaptic strengths were modified by every pair of spikes which occurred during a stimulus presentation, according to the rules for rSTDP and cSTDP. For cSTDP, the rule was
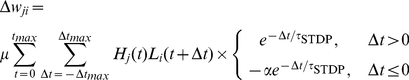
(5)


For rSTDP, the rule was
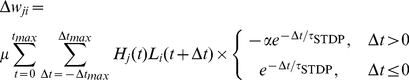
(5′)


We set 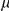 to 0.01, 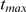 to 160 ms, and 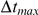 to 80 ms.

We stopped the simulations when either one of two conditions was reached:

1. If more than 50% of the top-down weights were within a distance of 0.1 of the maximum or minimum weights, we stopped the simulation and classified the outcome as ‘Extreme Weights’ (red bars in **Figures 5** and **6**).
2. If the cross-correlation between the current top-down weights and the weights of 3,000 stimulus presentations prior was greater than 0.99 *and* if the change in standard deviation of the distribution of top-down weights over the previous 6,000 presentations was less than 0.1% of the current value, we declared that the weights had stabilized, and stopped the simulation. If at this point the standard deviation of the weights was less than 0.3, we classified the outcome as ‘Weights too similar’ (blue bars in **Figures 5** and **6**). If, on the other hand, the standard deviation of the weights was greater than 0.3, we classified the outcome as ‘Convergent’ (green bars in **Figures 5** and **6**).

If neither stopping condition was reached after 625,000 stimulus presentations, we classified the simulation as ‘Did not converge’ (light blue bars in **Figures 5** and **6**). This last situation occurred in only a small fraction of the simulations.

We considered the parameters described in **Table 1** and ran a set of 6,912 simulations, to describe the conditions and sets of parameters for which learning would or would not converge. We varied 8 parameters, with 2–3 possible values for each parameter, and we considered all possible combinations. The results are summarized in **Figure 5**.

### Homeostatic synaptic scaling

For both the numerical implementation of the analytical work and the integrate-and-fire simulations, we made the same modifications to plasticity: after every 30 stimulus presentations, we calculated 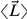, the vector of firing rates for every lower-level neuron averaged during those 30 presentations. We then applied the change 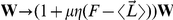 for a target firing rate *F* (the same for all neurons), where 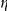 denotes a relative learning rate. This multiplied all the top-down inputs to a lower-level neuron by a constant value that is close to 1 when the neuron's firing is close to the target rate, or far from 1 otherwise. We also multiplied the strength of all future external inputs by an amount 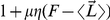. Taken together, these two changes were equivalent to changing the strength of all synaptic inputs to a lower-level neuron, both the bottom-up synapses carrying the external input and the top-down synapses carrying the feedback signal. We verified that this moved the firing rate towards the target value.

### Multiplicative scaling

We modified our learning rule to be dependent on the current weights, as follows. For the numerical implementation of the analytical model, we used
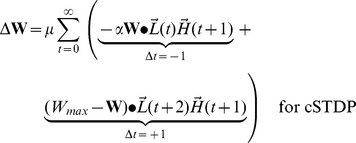
(6)

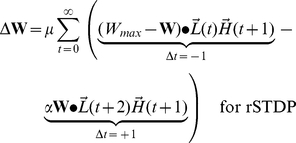
(6′)Here, the bullet represents entry-wise multiplication and 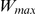 was varied among 3, 27, 30, and 60. For the integrate-and-fire simulations, we used
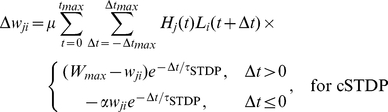
(7)

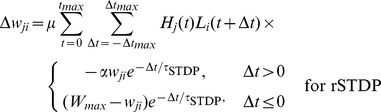
(7′)Here, 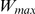 was the standard value used in the integrate-and-fire simulations, 50.

### Concurrent changes in Q

After each stimulus presentation, we applied the changes to 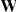 as usual by performing 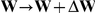. Additionally, we changed 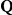. For the linear model, we used
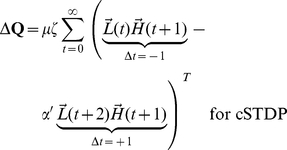
(8)

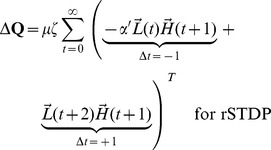
(8′)where 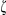 represented the relative strength of learning for top-down connections and 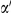 was the potentiation/depression bias for bottom-up connections, which could be different from that for top-down connections. We modified the learning rule for our integrate-and-fire simulations in the analogous way.

### Higher-layer external input

For the numerical simulations in **Figure 7**, we generated a cross-correlation matrix for higher-layer external inputs which was different from that for lower-layer external inputs. We modified our algorithm to include additional activity from the higher-layer inputs when calculating joint activity levels for learning by replacing **Equation 4** with the following equation, similar to **Equation 3**:
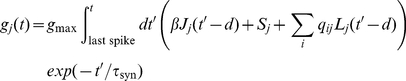
(9)


The external inputs 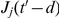 were generated in the same manner as the lower-layer inputs given in in **Equation 3**. In different simulations, we varied the strength of external higher-layer input 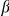 to be 0.1, 1, and 10 times the strength of the lower-layer input. We ran the simulations over the same 1,728 parameters used previously (but did not additionally run over the four combinations of different bottom-up and input cross-correlation matrixes).

### Measurement of reconstruction error

We modified the feedforward weight matrix **Q** by multiplying it by 2, subtracting the mean, and adding 0.5. Using this matrix, we trained the top-down weights as described previously. We then evaluated the ability of the top-down signals to provide a reconstruction of the original input at different time points after stimulus presentation and at different stages of training. We presented an early burst of external input to the network using a modified time-course that was zero after 50 ms. We measured the total number of spikes for each lower-level neuron during the first 50 ms, and separately during the time from 80–160 ms. We subtracted the later-time activity from that calculated in a network where the top-down weights were zero. Typically, in the absence of top-down weights, there was no later-time activity. We calculated the Pearson correlation coefficient between the vector of later-time mean activity levels with the vector of early-time activity level. We then repeated this procedure for *n* = 100 distinct external stimulus inputs, and averaged the correlation values. The results are shown in **Figure 8**. The correlation coefficients reached their maximum value and stabilized after stimulus presentations, so we used the weights at this time to generate the final activity (red line) in **Figure 8a**.

## Supporting Information

Text S1 Analytical formulation and analytical solutions.
(PDF)
