# Supplementary material for: Depression-Biased Reverse Plasticity Rule Is Required for Stable Learning at Top-Down Connections
Source: PLoS Comput Biol. 2012 Mar 1;8(3):e1002393. doi: 10.1371/journal.pcbi.1002393 (PMC3291526; doi:10.1371/journal.pcbi.1002393)
Supplement: Text S1 — Analytical formulation and analytical solutions. (PDF) [file pcbi.1002393.s001.pdf]

Supplementary Text

Network structure and neural activity

We consider the two-layer network described in **Figure 1a**. We denote the time-dependent activity levels in the two layers induced by a stimulus presentation as $\vec{L}(t)$ and $\vec{H}(t)$, respectively, where t represents the number of time-points since the beginning of the stimulus presentation. The evolution of activity in the network is determined by the synaptic weights: $\mathbf{Q}$ denotes the matrix representing the bottom-up weights and $\mathbf{W}(t)$ denotes the time-dependent matrix representing the top-down weights. We use t to denote the time since the beginning of the current stimulus presentation. As emphasized below, $\mathbf{W}$ also changes over the timescale of multiple stimulus presentations but we omit the stimulus presentation number here for simplicity in the notation. We assume linearity in the responses (this assumption is relaxed in the integrate-and-fire simulations, see main text). Neuronal activity is initialized at time $t = 0$ by an external stimulus $\vec{L}(0)$. At time $t = 1$, the higher level units are active as a result of the initial lower level activity according to $\vec{H}(1) = \mathbf{Q}\vec{L}(0)$. In the next time-point, the lower-level units are active as a result of the higher-level activity: $\vec{L}(2) = \mathbf{W}(1)\vec{H}(1)$, and so on. For later times, we can calculate the activity in the layers at each time point:

$$\begin{aligned}\vec{L}(2t) &= \Big[\prod_{t'=1}^{t}\big(\mathbf{W}(2t'-1)\mathbf{Q}\big)\Big]\vec{L}(0)\\ \vec{H}(2t+1) &= \mathbf{Q}\Big[\prod_{t'=1}^{t}\big(\mathbf{W}(2t'-1)\mathbf{Q}\big)\Big]\vec{L}(0)\end{aligned} \tag{A1}$$

Defining $\mathbf{W_0} \equiv \mathbf{W}(0)$, we approximate this activity as

$$\begin{aligned}\vec{L}(2t) &\approx \big(\mathbf{W_0}\mathbf{Q}\big)^t\vec{L}(0)\\ \vec{H}(2t+1) &\approx \mathbf{Q}\big(\mathbf{W_0}\mathbf{Q}\big)^t\vec{L}(0)\end{aligned} \tag{A2}$$

This will be a good approximation if a) activity decreases over time so that it approaches zero for $t \gg 1$, and b) learning is slow, so that any net change in $\mathbf{W}$ is negligible over the period when activity is large during a single stimulus presentation.

Synaptic plasticity in the weights is determined from the neural activities during the course of a stimulus presentation. For mathematical simplicity, we calculate the weight changes due to an infinite set of time-points following each stimulus presentation. The weight changes will be dominated by the effects of the early

time-points, so long as activity decreases over time. We consider joint activity in the two layers in pairs of adjacent time-points (in the integrate-and-fire simulations this simplification is relaxed, see main text). In our initial analyses, we fix $\mathbf{Q}$ and we study the changes in $\mathbf{W}$. (As discussed in the main text, this is consistent with studies that suggest that top-down connections develop after bottom-up ones; in **Figure 6** we explore the consequences of simultaneously changing $\mathbf{Q}$ and $\mathbf{W}$.) Focusing on the top-down connections, we consider both higher layer (pre-synaptic activity) preceding lower layer (post-synaptic) activity ($\Delta t = +1$) and post-synaptic activity preceding pre-synaptic activity ($\Delta t = -1$). The top-down weights evolve according to the following simplified version of spike-timing dependent plasticity (STDP) rule:

$$\Delta\mathbf{W} = \mu \sum_{t=0,2,4...}^{\infty} \big(\underbrace{-\alpha\vec{L}(t)\vec{H}(t+1)^{\intercal}}_{\Delta t=-1} + \underbrace{\vec{L}(t+2)\vec{H}(t+1)^{\intercal}}_{\Delta t=+1}\big) \quad \text{for cSTDP} \quad \text{(A3)}$$

$$\Delta\mathbf{W} = \mu \sum_{t=0,2,4...}^{\infty} \big(\underbrace{\vec{L}(t)\vec{H}(t+1)^{\intercal}}_{\Delta t=-1} - \underbrace{\alpha\vec{L}(t+2)\vec{H}(t+1)^{\intercal}}_{\Delta t=+1}\big) \quad \text{for rSTDP} \quad \text{(A4)}$$

where μ is the learning rate and α controls the relative bias of potentiation and depression and $^{\intercal}$ denotes the transpose operation. We note that this is a simplified and idealized version of STDP. Instead of weight changes that show an exponential dependence on Δt, here we use a step function and the weight changes only depend on activity in adjacent time points. A more realistic version of STDP is used in the integrate-and-fire simulations (see main text) but this idealized version captures the main aspects of STDP learning rules.

For simplicity, these two equations can be combined into the single equation

$$\Delta\mathbf{W} = \nu \sum_{t=0,2,4...}^{\infty} \big(\vec{L}(t)\vec{H}(t+1)^{\intercal} - \rho\vec{L}(t+2)\vec{H}(t+1)^{\intercal}\big) \quad \text{(A5)}$$

$$\nu, \rho = \begin{cases} -\mu\alpha, 1/\alpha & \text{for cSTDP} \\ \mu, \alpha & \text{for rSTDP} \end{cases} \quad \text{(A6)}$$

Here ρ represents the ratio of strengths of plasticity from pre-post synaptic spike pairs ($\Delta t = +1$) versus plasticity from post-pre synaptic spike pairs ($\Delta t = -1$).

We are interested in the evolution of the weights over many stimulus presentations. Let N represent the number of stimulus presentations. Plugging in the expressions for $\vec{L}(2t)$ and $\vec{H}(2t+1)$ into Equation (A5), we consider the

changes in $\mathbf{W}(N)$ in a single stimulus presentation, expressed as $d\mathbf{W}(N)/dN$. These changes depend on the external stimulation through the input activity L_0; here we average over all potential stimulus presentations by defining $\mathbf{C}_{L_0L_0} \equiv \langle \vec{L}(0)\vec{L}(0)^\intercal \rangle$, the cross-correlation of input stimuli, describing the average joint activities in the lower areas during the first time-point. We obtain the average weight change per stimulus presentation:

$$\begin{aligned}\frac{d\mathbf{W}}{dN} &= \nu(\mathbf{I} - \rho\mathbf{W_0Q})\sum_{t=0}^{\infty}(\mathbf{W_0Q})^t\langle \vec{L}(0)\vec{L}(0)^\intercal\rangle(\mathbf{Q}^\intercal\mathbf{W_0}^\intercal)^t\mathbf{Q}^\intercal \\ &= \nu(\mathbf{I} - \rho\mathbf{W_0Q})\sum_{t=0}^{\infty}(\mathbf{W_0Q})^t\mathbf{C}_{L_0L_0}(\mathbf{Q}^\intercal\mathbf{W_0}^\intercal)^t\mathbf{Q}^\intercal \qquad \text{(A7)}\end{aligned}$$

where $\mathbf{I}$ is the identity matrix.

$\rho > 1$ is required to prevent explosion in neuronal activity at fixed points

In order for the magnitude of weight changes given by our learning rule to be finite, we need activity in the network to decrease during the course of a stimulus presentation. If the network activity does not decrease over time, we can see that Equation (A1) and (A2) do not converge and lead to runaway excitation. More specifically, we see from Equations (A1) and (A2) that activity will decrease over time only if *all* eigenvalues of $\mathbf{W_0Q}$ have absolute value less than one. This condition is equivalent to requiring that the network have no strong excitatory loops.

We now suppose that $\mathbf{W}^*$ is a fixed point of the learning dynamics, such that $\frac{d\mathbf{W}}{dN}|_{\mathbf{W}=\mathbf{W}^*} = 0$. We define the matrix $\mathbf{X} \equiv \sum_{t=0}^{\infty}(\mathbf{W}^*\mathbf{Q})^t\mathbf{C}_{L_0L_0}(\mathbf{Q}^\intercal\mathbf{W}^{*\intercal})^t\mathbf{Q}^\intercal$. We thus have

$$\frac{d\mathbf{W}}{dN}\Big|_{\mathbf{W}=\mathbf{W}^*} = \nu(\mathbf{I} - \rho\mathbf{W}^*\mathbf{Q})\mathbf{X} = 0 \quad \Rightarrow \quad \mathbf{W}^*\mathbf{Q}\mathbf{X} = \frac{1}{\rho}\mathbf{X} \qquad \text{(A8)}$$

This is an eigenvalue equation, and it shows that $\mathbf{W}^*\mathbf{Q}$ has one or more eigenvalue equal to $1/\rho$. Because we have just argued that all eigenvalues of $\mathbf{W}^*\mathbf{Q}$ must have absolute value less than one, we conclude that our network can only have fixed points with finite activity when $\rho > 1$. This observation applies both for cSTDP and rSTDP. We recall that α was the parameter describing the relative strengths of depression and potentiation. For rSTDP, $\rho = \alpha > 1$ is the condition where depression dominates over potentiation. Conversely, for cSTDP, $\rho = 1/\alpha > 1$ is the condition where potentiation dominates.

Reverse STDP is required for development of unchanging top-down weights

By taking transposes of Equation (A8), and noting that $\mathbf{QX} = \mathbf{X}^\intercal\mathbf{Q}^\intercal$, we see that $\mathbf{QXW}^{*\intercal} = \frac{1}{\rho}\mathbf{X}^\intercal$, and thus that

$$(\mathbf{W}^*\mathbf{Q})\mathbf{X}(\mathbf{W}^{*\intercal}\mathbf{Q}^\intercal) = \frac{1}{\rho}\mathbf{W}^*\mathbf{X}^\intercal\mathbf{Q}^\intercal = \frac{1}{\rho^2}\mathbf{X} \tag{A9}$$

We then have

$$\begin{aligned}\frac{1}{\rho^2}\mathbf{X} = (\mathbf{W}^*\mathbf{Q})\mathbf{X}(\mathbf{W}^{*\intercal}\mathbf{Q}^\intercal) &= \sum_{t=0}^{\infty}(\mathbf{W}^*\mathbf{Q})(\mathbf{W}^*\mathbf{Q})^t\mathbf{C}_{L_0L_0}(\mathbf{Q}^\intercal\mathbf{W}^{*\intercal})^t\mathbf{Q}^\intercal(\mathbf{W}^{*\intercal}\mathbf{Q}^\intercal)\\ &= \sum_{t=0}^{\infty}(\mathbf{W}^*\mathbf{Q})^{t+1}\mathbf{C}_{L_0L_0}(\mathbf{Q}^\intercal\mathbf{W}^{*\intercal})^{t+1}\mathbf{Q}^\intercal\\ &= \sum_{t=1}^{\infty}(\mathbf{W}^*\mathbf{Q})^{t}\mathbf{C}_{L_0L_0}(\mathbf{Q}^\intercal\mathbf{W}^{*\intercal})^{t}\mathbf{Q}^\intercal \end{aligned} \tag{A10}$$

This is $\mathbf{X}$, minus the $t = 0$ term in the sum! Therefore,

$$(1 - \frac{1}{\rho^2})\mathbf{X} = \mathbf{C}_{L_0L_0}\mathbf{Q}^\intercal \tag{A11}$$

We thus see that $\mathbf{C}_{L_0L_0}\mathbf{Q}^\intercal$ is a scalar multiple of $\mathbf{X}$. From Equation A8, then, we know that

$$\mathbf{W}^*\mathbf{QC}_{L_0L_0}\mathbf{Q}^\intercal = \frac{1}{\rho}\mathbf{C}_{L_0L_0}\mathbf{Q}^\intercal \tag{A12}$$

and also that

$$(\mathbf{W}^*\mathbf{Q})\mathbf{C}_{L_0L_0}\mathbf{Q}^\intercal(\mathbf{W}^{*\intercal}\mathbf{Q}^\intercal) = \frac{1}{\rho^2}\mathbf{C}_{L_0L_0}\mathbf{Q}^\intercal \tag{A13}$$

When $\mathbf{QC}_{L_0L_0}\mathbf{Q}^\intercal$ is invertible, we can solve directly for the fixed point:

$$\mathbf{W}^* = \frac{1}{\rho}\mathbf{C}_{L_0L_0}\mathbf{Q}^\intercal(\mathbf{QC}_{L_0L_0}\mathbf{Q}^\intercal)^{-1} \tag{A14}$$

We now ask which types of networks can have *attractive* fixed points. We perform a linear stability analysis by considering the case where the top-down weights are a small distance $\mathbf{E}$ from a fixed point $\mathbf{W}^*$:

$$\frac{d\mathbf{E}}{dN} = \frac{d\mathbf{W}}{dN} = \nu(\mathbf{I} - \rho(\mathbf{W}^* + \mathbf{E})\mathbf{Q})\sum_{t=0}^{\infty}((\mathbf{W}^* + \mathbf{E})\mathbf{Q})^t\mathbf{C}_{L_0L_0}(\mathbf{Q}^\intercal(\mathbf{W}^* + \mathbf{E})^\intercal)^t\mathbf{Q}^\intercal \tag{A15}$$

We calculate the weight changes to first order in $\mathbf{E}$. The zeroth order terms disappear, using the result from A12. We are left with the first-order terms. We separate the parts which result from each of the three appearances of $\mathbf{E}$ in (A15) into three distinct terms, written here on three lines. We introduce the index t' to keep track of the different possible positions that $\mathbf{E}$ can take in the chains of multiplications which result from the expansions of $((\mathbf{W}^* + \mathbf{E})\mathbf{Q})^t$:

$$\begin{aligned}\frac{1}{\nu}\frac{d\mathbf{E}}{dN} \approx & -\rho\mathbf{E}\mathbf{Q}\sum_{t=0}^{\infty}(\mathbf{W}^*\mathbf{Q})^t\mathbf{C}_{L_0L_0}(\mathbf{Q}^\intercal\mathbf{W}^{*\intercal})^t\mathbf{Q}^\intercal \\ & + (\mathbf{I} - \rho\mathbf{W}^*\mathbf{Q})\sum_{t=1}^{\infty}\sum_{t'=0}^{t-1}(\mathbf{W}^*\mathbf{Q})^{t-t'-1}\mathbf{E}\mathbf{Q}(\mathbf{W}^*\mathbf{Q})^{t'}\mathbf{C}_{L_0L_0}(\mathbf{Q}^\intercal\mathbf{W}^{*\intercal})^t\mathbf{Q}^\intercal \\ & + (\mathbf{I} - \rho\mathbf{W}^*\mathbf{Q})\sum_{t=1}^{\infty}\sum_{t'=0}^{t-1}(\mathbf{W}^*\mathbf{Q})^t\mathbf{C}_{L_0L_0}(\mathbf{Q}^\intercal\mathbf{W}^{*\intercal})^{t'}\mathbf{Q}^\intercal\mathbf{E}^\intercal(\mathbf{Q}^\intercal\mathbf{W}^{*\intercal})^{t-t'-1}\mathbf{Q}^\intercal\end{aligned} \tag{A16}$$

The third term is zero, from Equation A12, because each multiplication of $\mathbf{C}_{L_0L_0}\mathbf{Q}^\intercal$ to the left by $\mathbf{W}^*\mathbf{Q}$ simply introduces a factor of $\frac{1}{\rho}$, which means the term in the parentheses is zero. The first term becomes

$$-\rho\mathbf{E}\mathbf{Q}\sum_{t=0}^{\infty}(\mathbf{W}^*\mathbf{Q})^t\mathbf{C}_{L_0L_0}(\mathbf{Q}^\intercal\mathbf{W}^{*\intercal})^t\mathbf{Q}^\intercal = -\rho\mathbf{E}\mathbf{Q}\sum_{t=0}^{\infty}\frac{1}{\rho^{2t}}\mathbf{C}_{L_0L_0}\mathbf{Q}^\intercal = \frac{-\rho}{1-\frac{1}{\rho^2}}\mathbf{E}\mathbf{Q}\mathbf{C}_{L_0L_0}\mathbf{Q}^\intercal \tag{A17}$$

Simplifying, again using Equation A12, we have

$$\begin{aligned}\frac{d\mathbf{E}}{dN} =& \nu\left[\frac{-\rho}{1-1/\rho^2}\mathbf{I} + (\mathbf{I} - \rho\mathbf{W}^*\mathbf{Q})\sum_{t=1}^{\infty}\sum_{t'=0}^{t-1}\frac{1}{\rho^{t+t'}}\left(\mathbf{W}^*\mathbf{Q}\right)^{t-t'-1}\right]\mathbf{E}\mathbf{Q}\mathbf{C}_{L_0L_0}\mathbf{Q}^\intercal \\ =& \left(\mathbf{Q}\mathbf{C}_{L_0L_0}\mathbf{Q}^\intercal \otimes \pm\nu\left[\frac{-\rho}{1-1/\rho^2}\mathbf{I} + (\mathbf{I} - \rho\mathbf{W}^*\mathbf{Q})\sum_{t=1}^{\infty}\sum_{t'=0}^{t-1}\frac{1}{\rho^{t+t'}}\left(\mathbf{W}^*\mathbf{Q}\right)^{t-t'-1}\right]\right)\mathbf{E}\end{aligned} \tag{A18}$$

The Jacobian matrix of our transformation, $\mathbf{J}$, is just the term in the parentheses. The fixed point $\mathbf{W}^*$ will be attractive if and only if the real parts of every eigenvalue in $\mathbf{J}$ are negative. The eigenvalues of a Kronecker product are the products of the eigenvalues of the components. Because $\mathbf{Q}\mathbf{C}_{L_0L_0}\mathbf{Q}^\intercal$ is positive semi-definite, it cannot have negative eigenvalues. Therefore, the sign of the eigenvalues of $\mathbf{J}$ will be determined by the eigenvalues of the bracketed term. If the bracketed term has only negative eigenvalues, the fixed point $\mathbf{W}^*$ will be attractive. If it contains any positive eigenvalues, $\mathbf{W}^*$ will be an unstable fixed point and learning will be unstable.

We know from Equation A8 that $\mathbf{W}^*\mathbf{Q}$ has at least one eigenvalue equal to $1/\rho$.

If we consider a corresponding eigenvector $\vec{V}$, we see that

$$\begin{aligned}
&\nu\big[\frac{-\rho}{1-1/\rho^2}\mathbf{I}+\sum_{t=1}^{\infty}\sum_{t'=0}^{t-1}\left(\mathbf{I}-\rho\mathbf{W}^*\mathbf{Q}\right)\frac{1}{\rho^{t+t'}}\left(\mathbf{W}^*\mathbf{Q}\right)^{t-t'-1}\big]\vec{V}\\
&\qquad=\nu\big[\frac{-\rho}{1-1/\rho^2}\mathbf{I}+\sum_{t=1}^{\infty}\frac{t}{\rho^{2t-1}}\left(\mathbf{I}-\rho\mathbf{W}^*\mathbf{Q}\right)\big]\vec{V}\\
&\qquad=\nu\frac{-\rho}{1-1/\rho^2}\vec{V} \qquad\qquad \text{(A19)}
\end{aligned}$$

Therefore $\nu\frac{-\rho}{1-1/\rho^2}$ is an eigenvalue of the bracketed term in Equation (A18). The sign of this quantity is equal to $-\text{sign}(\nu)$, since $\rho > 0$. Recall from our learning rule that ν is positive for rSTDP but negative for cSTDP. Therefore, for cSTDP, $\mathbf{J}$ will always have at least one positive eigenvalue and learning will always be unstable.

By contrast, learning with rSTDP can be stable (although it is not guaranteed to be.) For example, if $\mathbf{Q}$ is invertible, $\mathbf{W}^* = \mathbf{Q}^{-1}/\rho = \mathbf{Q}^{-1}/\alpha$ will be a fixed point; plugging this in to Equation (A18) confirms that it is an attractive fixed point. We show a numerical example of convergence to an attractive fixed point in **Figure 2**, and summarize these result in **Table 2**.

We have shown that stable learning in our network requires a depression-biased rSTDP rule.

For strong depression bias, learing minimizes reconstruction error

When the bias towards depression is strong, i.e. $\rho \gg 1$, Equation (A18) can be simply approximated:

$$\begin{aligned}
\frac{d\mathbf{E}}{dN}=\frac{d\mathbf{W}}{dN}&\approx\pm\,\nu\rho\mathbf{E}\left(\mathbf{Q}\mathbf{C}_{L_0L_0}\mathbf{Q}^\intercal\right)=\pm\nu\rho(\mathbf{W}-\mathbf{W}^*)\left(\mathbf{Q}\mathbf{C}_{L_0L_0}\mathbf{Q}^\intercal\right)\\
&=\pm\,\nu\rho\left(\mathbf{W}-\frac{1}{\rho}\mathbf{C}_{L_0L_0}\mathbf{Q}^\intercal(\mathbf{Q}\mathbf{C}_{L_0L_0}\mathbf{Q}^\intercal)^{-1}\right)\left(\mathbf{Q}\mathbf{C}_{L_0L_0}\mathbf{Q}^\intercal\right)\\
&=\pm\,\nu\left(\rho\mathbf{W}\mathbf{Q}-\mathbf{I}\right)\left(\mathbf{C}_{L_0L_0}\mathbf{Q}^\intercal\right) \qquad\qquad \text{(A20)}
\end{aligned}$$

We now define a *reconstruction error* as $\mathcal{E} = \langle||\vec{L}(0)-\rho\vec{L}(2)||^2\rangle_{\vec{L}(0)}$. A simple calculation shows that

$$\begin{aligned}
\frac{d\mathcal{E}}{d\mathbf{W}}&=\frac{d}{d\mathbf{W}}\langle||\vec{L}(0)-\rho\mathbf{W}\mathbf{Q}\vec{L}(0)||^2\rangle_{\vec{L}(0)}\\
&=2(\rho\mathbf{W}\mathbf{Q}-\mathbf{I})\langle\vec{L_0}\vec{L_0}^\intercal\rangle_{L\vec{(0)}}\mathbf{Q}^\intercal \qquad\qquad \text{(A21)}
\end{aligned}$$

Remembering that $\mathbf{C}_{L_0L_0} = \langle \vec{L_0}\vec{L_0}^\intercal \rangle_{\vec{L(0)}}$, we see that the approximate learning rule in (A20) performs gradient descent on the reconstruction error. This means that our depression-biased rSTDP learning rule attempts to find the set of feedback weights which do the best possible job at reconstructing the bottom-up input. When the feedforward weight matrix $\mathbf{Q}$ is invertible, the network is able to perfectly reproduce its input, and Equation (A14) becomes $\mathbf{W}^* = \mathbf{Q}^-1$. The current result tells us that even when the $\mathbf{Q}$ is not invertible – which will always be the case if there are fewer higher-layer neurons than lower-layer ones – the network will still move towards the best possible reconstruction.

Reformulation of the learning rule for easy numerical implementation

For ease of numerical implementation, we note that Equation (A7) can be simplified by taking the eigendecomposition of $\mathbf{W_0Q}$, finding a diagonal matrix $\mathbf{\Lambda}$ such that $\mathbf{W_0Q} = \mathbf{B\Lambda B^-1}$. Defining the additional diagonal matrix $\mathbf{K} \equiv \mathbf{\Lambda}\otimes\mathbf{\Lambda}$, we have

$$\begin{aligned}\frac{d\mathbf{W}}{dN} &= \nu(\mathbf{I} - \rho\mathbf{W_0Q})\mathbf{B}\big(\sum_{t=0}^{\infty}\mathbf{\Lambda}^t\mathbf{B^{-1}}\mathbf{C}_{L_0L_0}\mathbf{B^{-1}}^\intercal\mathbf{\Lambda}^t\big)\mathbf{B}^\intercal\mathbf{Q}^\intercal \\ &= \nu(\mathbf{I} - \rho\mathbf{W_0Q})\mathbf{B}\big(\sum_{t=0}^{\infty}\mathbf{K}^t\big)\mathbf{B^{-1}}\mathbf{C}_{L_0L_0}\mathbf{B^{-1}}^\intercal\mathbf{B}^\intercal\mathbf{Q}^\intercal \qquad \text{(A22)}\end{aligned}$$

Because $\mathbf{K}$ is diagonal, the sum can be easily evaluated. If the diagonal entries of $\mathbf{K}$ are $\{k_1, k_2, ...k_N\}$ and all have absolute values less than one, then $\sum_{t=0}^{\infty}\mathbf{K}^t$ is another diagonal matrix $\mathbf{K}'$ with entries $\{\frac{1}{1-k_1}, \frac{1}{1-k_2}, \cdots\frac{1}{1-k_N}\}$. We thus have:

$$\frac{d\mathbf{W}}{dN} = \nu(\mathbf{I} - \rho\mathbf{W_0Q})\mathbf{BK'B^{-1}}\mathbf{C}_{L_0L_0}\mathbf{Q}^\intercal \qquad \text{(A23)}$$

This formulation avoids infinite sums, thus allowing for numerical evaluation.

Changing Q while holding W fixed

We consider changing bottom-up connections rather than top-down ones, modifying $\mathbf{Q}$ while holding $\mathbf{W}$ constant. The analysis is very similar, with

$$\Delta\mathbf{Q} = \nu\sum_{t=0}^{\infty}\big(\vec{L}(t)\vec{H}(t+1) - \rho\vec{L}(t+2)\vec{H}(t+1)\big) \qquad \text{(A24)}$$

Note that that the identities of the pre-post versus post-pre neurons are reversed for bottom-up versus top-down connections, so the sign of learning is switched:

$$\nu, \rho = \begin{cases} \mu, \alpha & \text{for cSTDP} \\ -\mu\alpha, 1/\alpha & \text{for rSTDP} \end{cases} \tag{A25}$$

We again perform a linear stability analysis, expanding the learning rule to first order near a fixed point $\mathbf{Q}^*$:

$$\begin{aligned} \frac{1}{\nu}\frac{d\mathbf{E}}{dN} &= \frac{-\rho}{1-1/\rho^2}\mathbf{W}\mathbf{E}\mathbf{C}_{L_0L_0}\mathbf{Q}^{*\intercal} \\ &+ (\mathbf{I}-\rho\mathbf{W}\mathbf{Q}^*)\sum_{t=1}^{\infty}\sum_{t'=0}^{t-1}\left(\frac{1}{\rho^{t+t'}}(\mathbf{W}\mathbf{Q}^*)^{t-t'-1}\mathbf{W}\mathbf{E}\mathbf{C}_{L_0L_0}\mathbf{Q}^{*\intercal}\right) \\ &+ (\mathbf{I}-\rho\mathbf{W}\mathbf{Q}^*)\sum_{t=1}^{\infty}\left((\mathbf{W}\mathbf{Q}^*)^t\mathbf{C}_{L_0L_0}\mathbf{E}^\intercal(\mathbf{W}^\intercal\mathbf{Q}^{*\intercal})^t\right) \end{aligned} \tag{A26}$$

In the cases where $\mathbf{W}$ is invertible, we have $\mathbf{Q}^* = \frac{1}{\rho}\mathbf{W}^{-\mathbf{1}}$ and all terms but the first are zero:

$$\frac{d\mathbf{E}}{dN} = \nu\frac{-1}{1-1/\rho^2}\mathbf{W}\mathbf{E}\mathbf{C}_{L_0L_0}(\mathbf{W}^{-\mathbf{1}})^\intercal \tag{A27}$$

This is stable only when $\mathbf{W}$ has all positive or all negative eigenvalues and $\nu > 0$. For bottom-up neurons, $\nu > 0$ corresponds to cSTDP. Therefore, at least for invertible $\mathbf{W}$, bottom-up synapses must be trained with cSTDP to be stable.

Plasticity without reciprocal connections

We consider here the case where the lower and higher layers do not have feedforward connections between them. Instead, higher level neurons are activated at time $t = 1$ through an external stimulus. The initial activity in the lower level neurons is $\vec{L}_0$, and the activity in the higher level neurons is $\vec{H}$. Both $\vec{L}_0$ and $\vec{H}$ depend on the specific stimulus being presented. Because we do not have feedforward connections, the activity does not continue to reverberate past time $t = 2$, when activity in the lower level neurons is given by

$$\vec{L} = g(\mathbf{W}\vec{H}) \tag{A28}$$

Here, $g(x)$ is a potentially non-linear neural activation function which determines the firing rate of each lower level neuron given its summed synaptic input. We assume only that g rises monotonically, so that $g'(x) \geq 0$ for any x.

The learning rule becomes:

$$\Delta\mathbf{W} = \nu\big(\vec{L}_0\vec{H}^\intercal - \rho\vec{L}\vec{H}^\intercal\big) \tag{A29}$$

Averaging over many stimulus presentations, we have

$$\frac{d\mathbf{W}}{dN} = \nu\big(\langle\vec{L}_0\vec{H}^\intercal\rangle_{\text{stim}} - \rho\langle g(\mathbf{W}\vec{H})\vec{H}^\intercal\rangle_{\text{stim}}\big) \tag{A30}$$

We define $\mathbf{C}_{LH} \equiv \langle\vec{L}_0\vec{H}^\intercal\rangle_{\text{stim}}$, and $\mathbf{W}^*$ as the solution to $\mathbf{C}_{LH}/\rho = \langle g(\mathbf{W}^*\vec{H})\vec{H}^\intercal\rangle_{\text{stim}}$. We consider a weight matrix $\mathbf{E}$ which is very close to $\mathbf{W}^*$, so that we can approximate $g\big((\mathbf{W}^* + \mathbf{E})\vec{H}\big) \approx g(\mathbf{W}^*\vec{H}) + \mathbf{g}'\mathbf{E}\vec{H}$, where $\mathbf{g}' = \frac{dg(\vec{x})}{d\vec{x}}|_{\vec{x}=\mathbf{W}^*\vec{H}}$ is the diagonal Jacobian matrix of g evaluated at $\mathbf{W}^*\vec{H}$. We note that because g is a monotonically increasing function, $\mathbf{g}'$ is positive semi-definite.

$$\begin{aligned}\frac{d\mathbf{E}}{dN} &\approx -\nu\rho\langle\mathbf{g}'\mathbf{E}\vec{H}\vec{H}^\intercal\rangle_{\text{stim}} \\ &= \big(-\nu\rho\langle\vec{H}\vec{H}^\intercal \otimes \mathbf{g}'\rangle_{\text{stim}}\big)\mathbf{E}\end{aligned} \tag{A31}$$

We were able to move $\mathbf{E}$ outside the average over stimulus presentations because it is independent of the choice of stimulus. The Jacobian matrix of $\mathbf{E}$, $\mathbf{J}$, is just the term in the parentheses. The fixed point $\mathbf{W}^*$ will be attractive if and only if the real parts of every eigenvalue in $\mathbf{J}$ are negative. Because $\vec{H}\vec{H}^\intercal$ is positive semi-definite, the entire term inside the angle brackets is also positive semi-definite. Therefore, the sign of the eigenvalues of $\mathbf{J}$ are determined by the sign of $-\nu\rho$. Since we always have $\rho > 0$ and $\nu < 0$ for cSTDP, in this case we have only unstable fixed points. By contrast, rSTDP can have stable fixed points.
